# A Crosslinked HA-Based Hydrogel Ameliorates Dry Eye Symptoms in Dogs

**DOI:** 10.1155/2013/460437

**Published:** 2013-06-06

**Authors:** David L. Williams, Brenda K. Mann

**Affiliations:** ^1^Department of Veterinary Medicine, Madingley Road, Cambridge CB3 0ES, UK; ^2^SentrX Animal Care, Inc., 391 Chipeta Way, Suite G, Salt Lake City, UT 84108, USA; ^3^Department of Bioengineering, University of Utah, 72 S. Central Campus Drive, Rm. 2750, Salt Lake City, UT 84112, USA

## Abstract

Keratoconjunctivitis sicca, commonly referred to as dry eye or KCS, can affect both humans and dogs. The standard of care in treating KCS typically includes daily administration of eye drops to either stimulate tear production or to hydrate and lubricate the corneal surface. Lubricating eye drops are often applied four to six times daily for the life of the patient. In order to reduce this dosing regimen yet still provides sufficient hydration and lubrication, we have developed a crosslinked hydrogel based on a modified, thiolated hyaluronic acid (HA), xCMHA-S. This xCMHA-S gel was found to have different viscosity and rheologic behavior than solutions of noncrosslinked HA. The gel was also able to increase tear breakup time in rabbits, indicating a stabilization of the tear film. Further, in a preliminary clinical study of dogs with KCS, the gel significantly reduced the symptoms associated with KCS within two weeks while only being applied twice daily. The reduction of symptoms combined with the low dosing regimen indicates that this gel may lead to both improved patient health and owner compliance in applying the treatment.

## 1. Introduction

Keratoconjunctivitis sicca (KCS), commonly referred to as dry eye syndrome, is an ophthalmic disorder common in humans and dogs. The reported incidence of KCS in humans varies from 5 to 33%, depending on the report and how the data were obtained, while the incidence in dogs is approximately 1–4% [1]. In general, KCS results from a dysfunction in a component of the lacrimal functional unit, leading to changes in the volume, composition, or clearance of the tear film [2]. The lacrimal functional unit is composed of the lacrimal glands (both main and accessory), the ocular surface, and the interconnecting innervations [3]. In dogs, the most common cause of KCS is immune-related lacrimal gland disease [4, 5]. Other causes include congenital aplasia of the gland, drug-induced or traumatic injury to the gland, and neurologic dysfunction affecting the gland [4, 5]. With immune-mediated KCS in dogs, there is a predisposition for specific breeds having a higher prevalence. These breeds include English Bulldogs, West Highland White Terriers, Cavalier King Charles Spaniels, American and English Cocker Spaniels, and Pugs, with the prevalence reaching as high as 20% in these breeds [4, 5].

There is little information about the progression of dry eye symptoms in dogs with KCS as they age, but a gradual lowering of Schirmer tear test values in normal dogs has previously been shown as they age [6]. Although KCS is not considered a life-threatening disease, it often leads to corneal damage and scarring in the dog with concurrent, increased vascularization, hyperpigmentation, and vision loss. To treat KCS, surgery is sometimes required to transpose the parotid salivary duct into the ventral conjunctival sac allowing saliva to act as a tear replacement. However, KCS is more commonly treated by administering topical medications and hydrating drops daily for the life of the animal. Two common topicals are cyclosporine acting as a lacrimomimetic to stimulate increased tear production and a tear supplement to provide moisture and lubrication [4, 7]. Cyclosporine drops or ointment is often administered twice daily, although for difficult cases the dosing regimen may be more frequent [4]. The tear supplement, on the other hand, is often administered four times daily or more.

Tear supplements often contain a compound to increase the viscosity of the solution, such as polyvinyl alcohol, hydroxypropylmethylcellulose, carboxymethylcellulose, polyethylene glycol, or hyaluronic acid (HA) [4, 7, 8]. HA is particularly attractive as it is a naturally occurring polysaccharide, found throughout the body during all stages of development [9, 10]. In the eye, HA is in the aqueous humor and vitreous and also coats the corneal endothelium [11]. In tear supplements, the viscoelasticity of HA leads to a reduction of tear removal and an increase in tear stability, thereby reducing some of the symptoms of dry eye [12, 13].

Due to this viscoelasticity, one means of characterizing HA-based products is to determine the rheologic properties of the eye drop or gel. These properties are important to assess since the molecular weight and concentration of the HA will affect the viscosity, as well as elastic and viscous shear moduli of the solution [14, 15]. Additionally, the concentration of ions, such as salts, in the solution may influence the rheologic properties due to the polyanionic nature of HA [16, 17]. Several HA-based products with varying rheologic properties have been developed for ophthalmic surgery [18, 19], and these properties may influence comfort and efficacy in a dry eye formulation [14].

Although HA-based tear supplements have been used for over 20 years, a formulation that extends the contact time of the HA with the ocular surface may allow for less frequent application, reducing the overall cost and burden on the patient, and in the case of dogs, the owner. One method of extending the contact time may be to covalently crosslink the HA, rather than the physical or ionic crosslinking that occurs in solutions of high molecular weight HA. A modified HA, thiolated carboxymethyl HA (CMHA-S), has previously been used to create crosslinked hydrogels for treating skin and corneal wounds in multiple species [20, 21]. Here, we have developed a new hydrogel formulation as a tear supplement for treating KCS in dogs. The hydrogel was characterized using rheology to compare to noncrosslinked solutions of HA. The crosslinked CMHA-S hydrogel was then used in a clinical setting to treat dogs previously diagnosed with KCS, monitoring response to therapy by evaluating tear production (Schirmer tear test), conjunctival hyperaemia, ocular discharge, and ocular irritation as determined by blink frequency and palpebral aperture narrowing. With this new crosslinked hydrogel formulation, a reduction in both required application frequency and in KCS symptoms is anticipated and should improve both patient corneal health and owner compliance.

## 2. Materials and Methods

### 2.1. Crosslinked CMHA-S Hydrogel

CMHA-S was synthesized as previously described [21]. Thiol modification (3 × 10^−4^ mmol/mg) was assessed using 5,5′-dithio-bis(2-nitrobenzoic acid) (Ellman's reagent, Sigma-Aldrich). MW (340 kDa) was assessed using gel permeation chromatography and dynamic light scattering. Purified CMHA-S was diluted to a final concentration of 4 mg/mL in phosphate-buffered saline (PBS, pH 7.4, Fisher Scientific). The CMHA-S solution was then sterilized through a 0.2 *μ*m filter into a sterile mixing bowl. The solution was crosslinked using a dilute solution of sodium hypochlorite (Sigma-Aldrich) while mixing, and the resultant gel was mixed overnight. The crosslinked CMHA-S gel (xCMHA-S) was then packaged aseptically into sterile 15 mL eye drop bottles. A solution of noncrosslinked HA (900 kDa, Novozymes) at approximately 4 mg/mL in PBS (hereinafter referred to as HA4) was made for comparison in the rheological studies below.

To verify the final concentration of CMHA-S in the packaged xCMHA-S gel and HA in the noncrosslinked solution (HA4), a carbazole assay was used, which detects uronic acid [22]. The HA concentration in a commercial eye product that is a noncrosslinked solution of HA (Clinadry Eye Lubricant, IDPHAR Belgian Pharmaceuticals) (hereinafter referred to as HA2) was also assessed using this assay. Briefly, 250 *μ*L of sample or standard was combined with 1.5 mL ice-cold 0.025 M sodium tetraborate decahydrate (Sigma-Aldrich) in concentrated sulfuric acid (Acros Organics) and then heated in boiling water for 10 min, followed by rapid cooling in an ice bath. 50 *μ*L of 0.125% carbazole (Sigma-Aldrich) in analytical grade methanol (Acros Organics) was added to each sample and standard. The mixtures were heated in boiling water for 15 min and then cooled rapidly to room temperature. The absorbance at 530 nm was measured on a spectrophotometer (Genesys 10S, Thermo Scientific). Six samples were used for each formulation; standards used here were solutions of HA in PBS. 

### 2.2. Rheological Assessment

Rheological testing was performed on an AR1000 rheometer (TA Instruments) using a 25 mm diameter parallel plate format. Samples (5-6 mL) were placed in a 35 mm plastic Petri dish, and the rheometer head lowered to a gap distance of 5 mm. To determine viscosity, shear rate was varied from 0.1 to 100 s^−1^. A strain sweep was then performed at an oscillation frequency of 1 Hz, varying strain from 0.005 to 0.5 to determine the limits of the linear viscoelastic region. From these strain sweeps, a strain of 0.03 was used for subsequent frequency sweeps as it fell within the linear region for all samples tested. The elastic modulus, *G*′, and viscous modulus, *G*′′, were then measured at a constant strain of 0.03 over a frequency range of 0.05 to 10 Hz. The ratio of *G*′′/*G*′ is called the loss tangent, or tan*δ*, and is a measure of the ratio of the energy lost to the energy stored in deformation [23].

### 2.3. Tear Breakup Time in Rabbits

The experimental protocol and animal care complied with the NIH Guide for the Care and Use of Laboratory Animals was approved by the Institutional Animal Care and Use Committee for the University of Utah. Tear breakup time (TBUT) was assessed by administering fluorescein dye to the eye and visualizing the tear film using the cobalt blue function of a slit lamp microscope. TBUT is the time that elapses between the last blink and the first appearance of breakup in the fluorescein and is an indicator of tear film stability [24]. 

A total of six New Zealand White Rabbits were utilized in this study. Two animals were used as negative controls, and 5 *μ*L of fluorescein sodium benoxinate hydrochloride ophthalmic solution USP 0.25%/0.4% (Bausch & Lomb) was administered with a micropipette to the corneal surface. After administration, the lid was closed manually twice to distribute the tear film and agent. The eye was then gently held open, and the TBUT was measured and recorded. Both eyes of each animal were used for the negative controls. The TBUT was scored by two individuals agreeing and confirming the time. In all cases, both individuals who were scoring were masked to which active compound was being installed.

The following day, three animals each received either xCMHA-S gel or HA2 drops (used as a positive control). For the xCMHA-S and HA2, 500 *μ*L of the material was mixed with 2 drops (100 *μ*L total) of the fluorescein solution to create a homogeneous mixture. Although addition of the fluorescein solution may have reduced the apparent viscosity of each material, the amount of fluorescein used was necessary for visualization with the slit lamp. 50 *μ*L of the mixture was then applied to the corneal surface using a micropipette. The lid was blinked manually twice as with the negative control and then gently held open to record TBUT.

### 2.4. Clinical Evaluation in Dogs with KCS

25 dogs with KCS were included in the study, as detailed in Table 1. The canine patients were referred to the Queen's Veterinary School Hospital, Department of Veterinary Medicine, University of Cambridge, where a diagnosis of KCS was confirmed. All animals were examined by direct and indirect ophthalmoscopy and slit lamp biomicroscopy. Ocular surface health was determined, as characterized by changes in ocular hyperaemia, ocular irritation, and ocular discharge; each of these clinical features scored as normal (0), mildly impaired (1), or severely impaired (2). Tear production was assessed using the Schirmer tear test (STT) in which normal canine eyes yield strip wetting of between 15 and 20 mm in one minute, but eyes with strip wetting of less than 10 mm in one minute are classified as being mildly affected by KCS, and those with strip wetting of less than 5 mm/min are considered severely affected. Dogs were reexamined after two weeks of twice daily treatment with the xCMHA-S gel, and assessments of ocular surface health were repeated. Data were compared with those from a previous study of 25 dogs treated with HA2 drops [25].

### 2.5. Statistical Analysis

Reported *P* values were determined using unpaired, two-tailed Student's *t*-tests between materials for both rheological studies and the TBUT study. Mann-Whitney *U* tests were used to compare the categorical score data on improvements in ocular surface health comparing results before and after treatment, as well as comparing treatment with the xCMHA-S gel and HA2 drops. Averages are reported as mean ± standard deviation.

## 3. Results

### 3.1. Rheological Assessment

To determine the effect of covalently crosslinking modified HA on material properties, the viscosity and shear moduli of the xCMHA-S gel and a noncrosslinked solution of HA at a similar concentration (HA4) were determined. These were compared to a commercial eye product (HA2) that is also a noncrosslinked solution of HA but at a lower concentration (see Table 2). 

**Figure 1:**
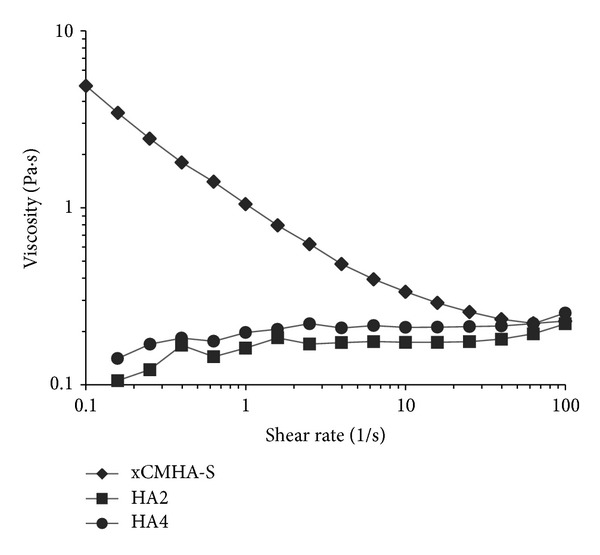
Steady shear viscosity of the crosslinked CMHA-S gel (xCMHA-S) and noncrosslinked HA solutions (HA2 and HA4) as a function of shear rate.

Steady shear viscosities of the three materials as a function of shear rate are shown in Figure 1. There is a distinct difference in the behavior of the xCMHA-S gel compared to HA2 and HA4 and the noncrosslinked HA solutions. The viscosity of the xCMHA-S gel decreases with increasing shear rate, indicating a shear thinning behavior. The noncrosslinked HA solutions, on the other hand, do not shear thin but rather display Newtonian behavior. Additionally, the viscosity of the xCMHA-S gel at shear rates of 0.25 and 2.5 s^−1^ is significantly greater than the viscosities of the noncrosslinked HA solutions (see Table 2).

**Figure 2:**
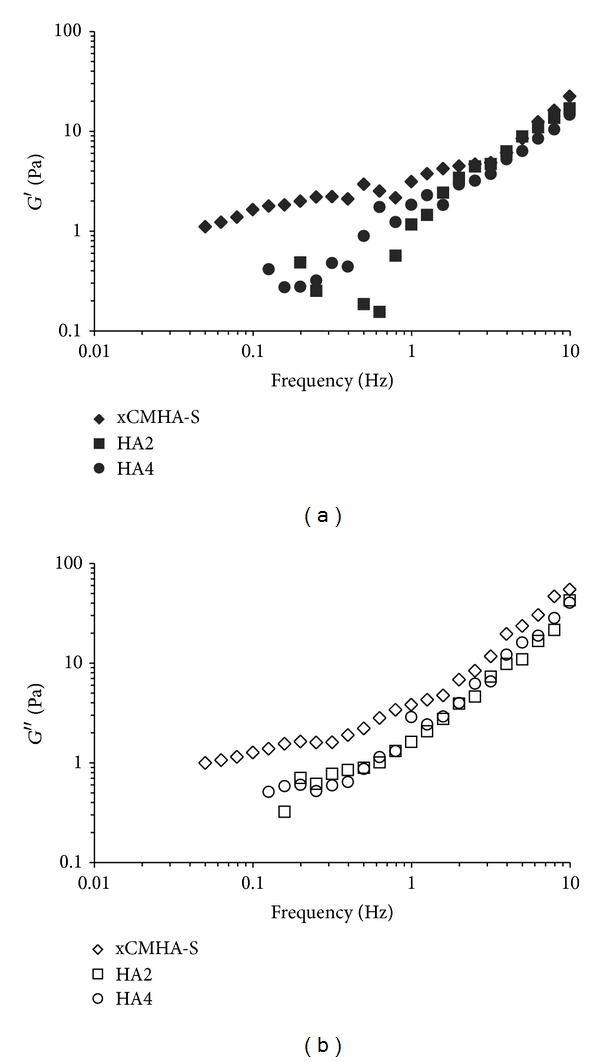
(a) Elastic modulus, *G*′, and (b) viscous modulus, *G*′′, of the crosslinked CMHA-S gel (xCMHA-S) and noncrosslinked HA solutions (HA2 and HA4) as a function of frequency.

The elastic, *G*′, and viscous, *G*′′, moduli as a function of frequency are shown in Figures 2(a) and 2(b), respectively. At low frequencies, below about 1 Hz, the xCMHA-S gel has a significantly greater elastic modulus than the other two materials. However, at higher frequencies, their elastic moduli are similar. This is also seen in Table 2 when comparing the elastic moduli for each material at 2.5 Hz. The viscous modulus, on the other hand, is always greater for the xCMHA-S gel than the noncrosslinked HA solutions, illustrated in Table 2 for *G*′′ at 2.5 Hz. Additionally, for the xCMHA-S gel, at low frequencies, *G*′ and *G*′′ are very similar, whereas at frequencies above approximately 2 Hz, *G*′′ > *G*′. For the noncrosslinked HA solutions, *G*′′ > *G*′ over much of the frequency range, except between about 1 and 5 Hz for HA2 and about 0.3 and 2 Hz for HA4, when *G*′ and *G*′′ are roughly equal. Loss tangents for these materials at 0.5 and 5 Hz are also provided in Table 2.

### 3.2. Tear Breakup Time in Rabbits

TBUT was assessed with fluorescein and slit lamp evaluation. The xCMHA-S gel developed here was compared to a positive control (HA2) and a negative control (fluorescein alone). The application of xCMHA-S gel drops increased TBUT compared to HA2 (93 ± 12 sec and 71 ± 7 sec, resp.), although the difference was not statistically significant (*P* = 0.056, *n* = 3). Both of these treatments resulted in significantly longer TBUTs compared to negative control (38 ± 2 sec, *P* < 0.0005 for each). TBUTs were evaluated after only one application of each material. Upon application of all study agents, eyes were both white and quiet. After thorough and repeated observation at 24 hours postprocedure, no eyes showed any sign of irritation or intolerability to any of the applied study agents.

### 3.3. Clinical Evaluation in Dogs with KCS

The mean STT readings before and after two weeks of treatment for the 25 dogs on the xCMHA-S gel are given in Table 1 together with scores for conjunctival hyperaemia, ocular irritation, and ocular discharge. The mean STT readings were not significantly changed from pre- to posttreatment; however, categorical scores of ocular surface health were significantly improved posttreatment. When compared with values previously reported for HA2 [25], improvements in conjunctival hyperaemia, ocular irritation, and ocular discharge were significantly better for the xCMHA-S gel, with *P*-values of 0.016 and 0.006 for conjunctival hyperaemia in the right and left eyes, respectively, 0.052 and 0.053 for irritation in right and left eyes, and 0.02 and 0.08 for ocular discharge, again in right and left eyes, respectively.

## 4. Discussion

KCS is a disease that requires lifelong treatment to mitigate potential corneal damage and vision loss, which often includes the application of a tear supplement for moisture and lubrication. Currently available tear replacement medications often have to be applied several times daily. A longer acting tear supplement that could subsequently be applied less frequently may be beneficial for both patients and owners of pets with KCS. Here, we have developed an HA-based crosslinked gel as an extended KCS treatment. We characterized the viscosity and shear moduli of the gel and compared them to two different solutions of noncrosslinked HA. We also determined the effect of applying the xCMHA-S gel to the cornea on tear breakup time. We then used the xCMHA-S gel in a clinical setting, treating dogs diagnosed with KCS.

Due to the viscoelastic nature of HA, as well as the fact that it is anionic, it is important to assess the rheologic properties of HA-based materials. In solutions of HA, the molecular weight, concentration of HA, and the presence of salts or other charged molecules such as proteins can affect the viscosity and shear moduli. Additionally, covalently crosslinking the HA to form hydrogels can further impact these rheologic properties. The xCMHA-S gel developed here is made using a modified HA, in which two modification have been made. The first modification attaches carboxyl groups to some of the hydroxyl groups. The second modification attaches thiol groups to some of the carboxyl groups. These thiol groups are then utilized for the covalent crosslinking, forming disulfide bonds. We have therefore not only crosslinked the modified HA but also have modified the ionic nature of the molecule as well. Further, it should be noted that during the crosslinking process, the material is continuously mixed using a whisk attachment with a planetary mixer. This results in a material that is not a single continuous gel but rather appears to be effectively a collection of microgels based on both its physical appearance and its rheological properties. 

The xCMHA-S gel was found to have a steady shear viscosity that decreased with increasing shear rate, typical of other hydrogels that display shear thinning (pseudoplastic) properties [26]. It is also similar to the viscosity behavior observed with methacrylated HA near gels and microgels [27], as well as HA-containing materials in the body, including synovial fluid and the vitreous [28, 29]. The ability to shear thin may be particularly important for comfort on the corneal surface, allowing the gel to thin during a blink. This behavior was not seen with either the commercial eye lubricant (HA2), a solution of HA at about 0.2%, nor with another HA solution (HA4) at about 0.4%. These solutions displayed typical Newtonian behavior, with a roughly constant viscosity over much of the range of shear rates used here, similar in behavior to previous results for HA solutions with low concentrations [27, 30]. Although the molecular weight of the HA used in HA2 was not determined, it is likely much higher than the HA used for HA4 here (900 kDa) and the HA used in a previous study (1.6 MDa) [27] based on their viscosities. Additionally, given the fact that the xCMHA-S gel and the HA4 had roughly the same concentration (about 0.4%) in the same buffer (PBS), the change in behavior for the viscosity can be attributed primarily to the crosslinked nature of the xCMHA-S gel. Although it might have been interesting to compare the xCMHA-S gel to a noncrosslinked solution of the CMHA-S in order to have the same molecular weight and ionic properties, this would have been very difficult to achieve and test, given that the disulfide crosslinking occurs in the presence of oxygen at the pH used here. Thus, we would have had to either remove the dissolved oxygen from the solution and perform the rheological testing under argon or nitrogen or lower the pH which would have changed ionic interactions.

In addition to determining the viscosity of the materials, we evaluated the elastic and viscous shear moduli under constant strain. It is important to evaluate these materials under conditions of small-amplitude oscillation as well, since the steady-state viscosity determinations at high shear rates can lead to distortion of a large molecule, such as HA, and structural breakdown of a gel [31]. For the xCMHA-S gel, the elastic component is slightly greater than the viscous component at low frequencies; however, at high frequencies, the material behaves more as a viscous solution. This is highlighted by the change in the loss tangent from 0.7 to 2.8 at frequencies of 0.25 and 5 Hz, respectively. A very similar rheological behavior was observed for methacrylated HA microgels at the higher volume fractions used [27] and poly-NIPAAm microgels [32]. Other crosslinked HA gels have displayed more elastic behavior, with *G*′ > *G*′′ over the same range of frequencies [26, 29]; however, these gels typically have a higher concentration of HA and a higher crosslink density than the xCMHA-S gel developed here. The HA solutions, on the other hand, demonstrated a predominantly viscous behavior throughout much of the frequency range, with tan⁡*δ* > 1 at both high and low frequencies for each solution. A similar behavior has been found for other HA solutions at low concentration or with lower molecular weight [28, 30].

TBUT is a standard measure employed to evaluate topical dry eye supplements and the stability of tear film on the ocular surface [33]. Both the xCMHA-S gel and HA2 extended the TBUT compared to a negative control, indicating that both are able to stabilize the tear film. Further, the xCMHA-S gel appeared to have a greater effect on the corneal tear film compared to HA2. Although the *P*-value (0.056) did not reach the level of statistical significance, this was likely due to the low number of animals used (*n* = 3), and the results suggest a potential benefit of using a crosslinked gel to treat KCS. Additionally, the rabbits used for this study had a normal tear film and thus were not a dry eye disease model. Nevertheless, the results of this study correspond well with previous studies that indicate the presence of HA which stabilizes the corneal tear film [34–36], and these results indicate that a crosslinked form may enhance this more than a noncrosslinked form. It is not clear from the present study, however, whether the enhanced effect is due to the differences in viscosity or rheological properties found between the xCMHA-S gel and the noncrosslinked HA solutions or some other mechanisms.

With the promising results observed in the rheological assessment and TBUT study for the xCMHA-S gel, we wanted to determine whether this formulation could be used only twice daily in a clinical setting and result in improvements in the symptoms associated with KCS in dogs. The xCMHA-S gel significantly improved the characteristic signs of canine keratoconjunctivitis sicca in two weeks when given on this dosing schedule, a reduced dosing schedule compared to many other tear supplements [4, 36]. Although the STT value did not change from pre- to posttreatment, we did not expect it to since the material, acting as a tear supplement, should not stimulate an increase in tear production itself but rather improve the symptoms associated with KCS. Comparison with a previous study [25] shows that the improvements seen with the crosslinked modified HA are significantly better than with a standard noncrosslinked solution of HA (HA2). Clearly, there may be potential problems with comparing two different groups of dogs; however, the pretreatment STT values and scores for conjunctival hyperaemia, ocular irritation, and ocular discharge were not significantly different (*P* always >0.05) between the groups of dogs, suggesting that the two groups were similar enough for valid comparisons to be made. We also recognize that the current clinical study was unmasked and therefore could have led to some bias, yet we consider these very promising results. A blinded study is currently underway directly comparing the two tear replacement formulations in KCS-affected dogs.

We have produced a crosslinked HA-based hydrogel formulation intended to be used in relieving the symptoms of KCS. This crosslinked version of HA has significantly different viscosity and rheological properties than corresponding noncrosslinked solutions of HA. Additionally, this gel is able to stabilize the tear film, potentially better than a simple solution of HA, and was able to lead to improvements in KCS symptoms in dogs in a preliminary clinical setting within two weeks despite being used only twice daily. Such a formulation may lead to better patient eye health and owner compliance. This formulation not only has relevance for the pet dog population but also uses these animals as a naturally occurring spontaneous model for human dry eye, a significant problem where the drive for better tear replacements continues apace.

## Figures and Tables

**Table 1:** Scores of ocular health before and after 2 weeks of treatment (twice daily) of the xCMHA-S gel in a clinical study on dogs.

Case	Breed	Age (yrs)/Gender	STT before (mm/min)	STT after (mm/min)	Hyperaemia Before	Hyperaemia After	Irritation Before	Irritation After	Discharge Before	Discharge After
1	CKCS	8/Fn	2/4	3/3	2/2	1/1	2/2	0/0	1/1	0/0
2	Bulldog	6/Fn	10/9	6/2	2/2	0/0	1/1	0/0	2/2	0/0
3	Crossbred	10/Fn	8/4	9/6	2/1	1/0	2/0	0/0	2/1	0/0
4	St. Poodle	11/Fn	7/4	6/4	2/2	0/0	2/1	0/0	1/1	0/0
5	CKCS	6/Fn	2/2	3/3	1/1	0/0	1/1	0/0	0/0	0/0
6	CKCS	8/Fn	3/3	3/4	2/2	0/0	2/2	0/0	1/1	0/0
7	Shih Tzu	12/Fn	2/1	3/1	2/2	0/0	2/2	0/0	2/2	1/1
8	Shih Tzu	8/Fe	0/1	2/2	2/2	1/1	2/2	0/0	1/1	0/0
9	Bassett	7/Mn	4/9	2/8	2/2	1/1	2/2	0/0	0/0	0/0
10	WHWT	12/Fn	2/1	4/3	2/2	1/1	2/2	1/1	1/1	0/1
11	Lhasa Apso	11/Fe	3/7	2/6	1/1	0/0	1/1	0/0	0/0	0/0
12	ECS	10/Mn	3/2	3/2	2/2	0/0	1/1	0/0	1/1	0/0
13	Eng Setter	7/Mn	8/7	7/7	1/1	0/0	1/1	0/0	0/0	0/0
14	Min Schn	8/Fn	9/8	8/7	1/1	0/0	2/2	0/0	0/0	0/0
15	ECS	9/Fe	3/5	2/5	1/1	0/0	1/1	0/0	0/0	0/0
16	ECS	8/Fn	3/7	2/6	2/2	0/1	2/2	0/0	1/1	0/0
17	WHWT	13/Fn	0/1	2/2	2/2	0/0	2/2	0/0	2/2	1/1
18	WHWT	5/Fe	0/0	1/2	1/1	0/0	1/1	0/0	1/1	0/0
19	Rottweiler	8/Me	3/8	4/9	2/2	1/1	2/2	1/1	0/0	0/0
20	Shih Tzu	7/Fn	4/7	5/8	2/1	1/1	1/1	0/0	0/0	0/0
21	Weimaraner	8/Mn	9/9	8/12	2/2	1/1	2/2	0/0	0/0	0/0
22	ECS	10/Fn	4/5	4/5	2/2	0/0	2/2	1/1	0/0	0/0
23	ECS	12/Fn	5/5	3/6	1/1	0/0	1/1	0/0	0/0	0/0
24	Shih Tzu	7/Me	5/6	6/6	2/2	0/0	1/1	1/1	1/1	0/0
25	Shih Tzu	9/Fn	4/4	5/4	2/2	0/0	2/2	1/1	0/0	0/0
Mean ± SD			4.12 ± 2.88/ 4.76 ± 2.86	4.12 ± 2.22/ 4.92 ± 2.68	1.70 ± 0.44/ 1.68 ± 0.48	0.28 ± 0.46/ 0.24 ± 0.44	1.60 ± 0.50/ 1.50 ± 0.59	0.16 ± 0.37/ 0.12 ± 0.33	0.76 ± 0.78/ 0.96 ± 1.62	0.08 ± 0.28/ 0.06 ± 0.18
*P* value				1.00/0.67		4.3*E* − 12/ 6.6*E* − 11		6.9*E* − 12/ 1.4*E* − 10		5.0*E* − 05/ 1.1*E* − 02

All scores given are for right eye/left eye and indicate normal (0), mildly impaired (1), or severely impaired (2).  *P* values indicate comparison between scores before and after treatment. In breed: CKCS: Cavalier King Charles Spaniel; St.: Standard; WHWT: West Highland White Terrier; ECS: English Cocker Spaniel; Eng: English; Min Schn: Miniature Schnauzer. In Gender: Fn: neutered female; Fe: unaltered female; Mn: neutered male; Me: unaltered male. STT: Schirmer tear test.

**Table 2:** Properties of the three materials tested in this study. The concentration of CMHA-S or HA was determined usin a carbazole assay (*n* = 6). Viscosity and shear moduli are averages of 8 samples.

Property	xCMHA-S	HA2	HA4
CMHA-S or HA conc. (mg/mL)	3.77 ± 0.09	2.50 ± 0.03	4.35 ± 0.06
Viscosity at 0.25 s^−1^ (Pa·s)	2.82 ± 0.50	0.09 ± 0.03	0.14 ± 0.03
Viscosity at 2.5 s^−1^ (Pa·s)	0.66 ± 0.06	0.17 ± 0.01	0.21 ± 0.01
*G*′ at 2.5 Hz (Pa)	4.14 ± 1.23	4.46 ± 1.47	3.25 ± 1.19
*G*′′ at 2.5 Hz (Pa)	8.75 ± 1.21	4.37 ± 0.70	6.39 ± 1.80
tan *δ* at 0.25 Hz	0.7	2.4	1.6
tan *δ* at 5 Hz	2.8	1.2	2.5
